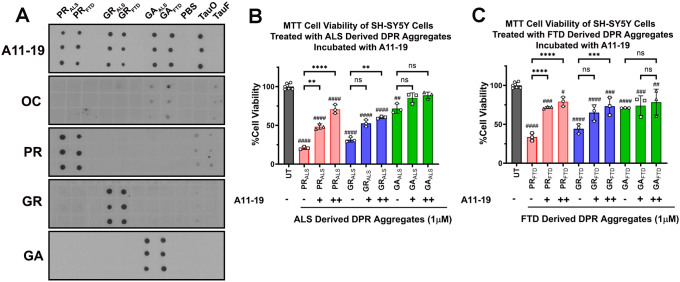# *C9orf72*-associated dipeptide protein repeats form A11-positive oligomers in amyotrophic lateral sclerosis and frontotemporal dementia

**DOI:** 10.1016/j.jbc.2024.105628

**Published:** 2024-01-10

**Authors:** Nemil Bhatt, Nicha Puangmalai, Urmi Sengupta, Cynthia Jerez, Madison Kidd, Shailee Gandhi, Rakez Kayed

**Affiliations:** 1 Mitchell Center for Neurodegenerative Disease, University of Texas Medical Branch, Galveston, Texas, USA; 2 Department of Neurology, Neuroscience and Cell Biology, University of Texas Medical Branch, Galveston, Texas, USA

**Keywords:** dipeptide protein repeats, amyloids, ALS, FTD, neurodegeneration, C9orf72

## Abstract

Hexanucleotide repeat expansion in C9orf72 is one of the most common causes of amyotrophic lateral sclerosis and frontotemporal dementia. The hexanucleotide expansion, formed by GGGGCC (G4C2) repeats, leads to the production of five dipeptide protein repeats (DPRs) *via* repeat-associated non-AUG translation. Among the five dipeptide repeats, Gly-Arg, Pro-Arg, and Gly-Ala form neuronal inclusions that contain aggregates of the peptides. Several studies have attempted to model DPR-associated toxicity using various repeat lengths, which suggests a unique conformation that is cytotoxic and is independent of the repeat length. However, the structural characteristics of DPR aggregates have yet to be determined. Increasing evidence suggests that soluble species, such as oligomers, are the main cause of toxicity in proteinopathies, such as Alzheimer’s and Parkinson’s disease. To investigate the ability of DPRs to aggregate and form toxic oligomers, we adopted a reductionist approach using small dipeptide repeats of 3, 6, and 12. This study shows that DPRs, particularly glycine–arginine and proline–arginine, form oligomers that exhibit distinct dye-binding properties and morphologies. Importantly, we also identified toxic DPR oligomers in amyotrophic lateral sclerosis and frontotemporal dementia postmortem brains that are morphologically similar to those generated recombinantly. This study demonstrates that, similar to soluble oligomers formed by various amyloid proteins, DPR oligomers are toxic, independent of their repeat length.

Amyotrophic lateral sclerosis (ALS) is clinically characterized by voluntary motor deficits that lead to muscle weakness, whereas frontotemporal dementia (FTD) is characterized by selective degeneration in the frontal and temporal cortex leading to dementia among other neurological symptoms (1, 2, 3). Despite their distinct clinical manifestations, both ALS and FTD have protein aggregates as their hallmark (4, 5). Moreover, these diseases belong to the same clinicopathological spectrum and share overlapping protein pathologies as well as genetic mutations (1). GGGGCC (G4C2) intronic hexanucleotide repeat expansion (HRE) in the first intron of C9orf72 is the most common inherited cause of both ALS and FTD (6). This mutation explains up to 40% of familial cases in ALS and FTD (6). Carriers of this mutation tend to have tens to hundreds of G4C2 repeats, whereas healthy individuals have no more than two to eight repeats (7). However, the correlation between repeat length and disease severity is yet to be established (3).

Since the discovery of C9orf72 HRE, several hypotheses have been proposed to explain the link between the mutation and disease. Strong evidence for gain-of-toxic function has emerged. For example, the sense and antisense RNA generated from HRE accumulate and form G-quadruplex structures (8, 9). The sense and antisense RNA are shown to cause toxicity in several different animal models (9, 10). However, phase 1 clinical trial targeting the sense RNAs using antisense oligonucleotides was terminated because of lack of efficacy, suggesting that the disease mechanism is much more complex (11). An alternative gain-of-function theory is that the sense and antisense RNA are translated by repeat-associated non-ATG translation (RAN) (3, 10). The RAN-translated proteins are dipeptide repeats of glycine–alanine (GA), glycine–arginine (GR), proline–alanine, proline–arginine (PR), or glycine–proline (GP) (12). The dipeptide protein repeats (DPRs) are reported in tissues of C9orf72 HRE-positive patients, suggesting their pathological role (13). Moreover, DPRs aggregate and follow the amyloid cascade (14, 15). They form inclusions and then sequester and aggregate other proteins, which may trigger the disease onset (15, 16, 17, 18, 19, 20, 21). DPR inclusions have been identified in the hippocampus, frontal cortex, cerebellum, motor cortex, as well as the basal ganglia in patients with ALS/FTD (1, 13, 22, 23, 24, 25).

Of the five DPRs, GA is most readily visible in inclusions in the brain and spinal cord of patients with ALS and FTD. GA has a shown propensity to aggregate and form amyloidogenic fibrils made from parallel β-sheet structures, consistent with amyloid-β in Alzheimer’s disease (15). Although there is a consensus that GA is toxic when expressed at high levels, it is less toxic than arginine containing DPRs like PR and GR (16, 17, 21). The exact mechanism of GR and PR toxicity is still unclear; however, it has been hypothesized that GR and PR aggregate slowly, whereas GA rapidly aggregates to form nontoxic insoluble fibrillar structures (16, 26).

A vast number of studies have shown that soluble oligomers of amyloid-β, α-synuclein, tau, and TDP-43 (TAR DNA-binding protein 43) are the toxic species that appear before disease onset and are one of the main drivers of other neurodegenerative diseases (14, 27, 28, 29, 30, 31). Despite there being a plethora of studies investigating DPRs in ALS/FTD and their associated toxicity *in vitro*, the structural characteristics of DPR aggregates in ALS/FTD have yet to be determined. A study conducted by Flores *et al*. (32)revealed that three DPRs (GR, GP, and GA) form distinct structures, regardless of the length of repeats. In addition, the results showed that the DPRs were internalized. Their work, along with several other studies, has investigated the DPR-associated toxicity using various repeat lengths, showing most repeat lengths are toxic, suggesting unique conformation that mediates toxicity independent of DPR length (16, 17, 21, 33). Moreover, RAN translation does not produce DPRs of uniform length; rather, DPRs are translated in a combination of repeats because of ribosomal frameshifting (34, 35, 36, 37). To address critical gaps in understanding of C9orf72-associated DPR structures and their toxicity, we sought to determine whether DPRs, particularly GR, PR, and GA (reported to be the most toxic in the literature), form distinct cytotoxic soluble oligomers in ALS and FTD as they are found similarly in other neurodegenerative diseases.

## Results

### Arginine containing DPRs form A11–19 positive oligomers

**Figure 1:**
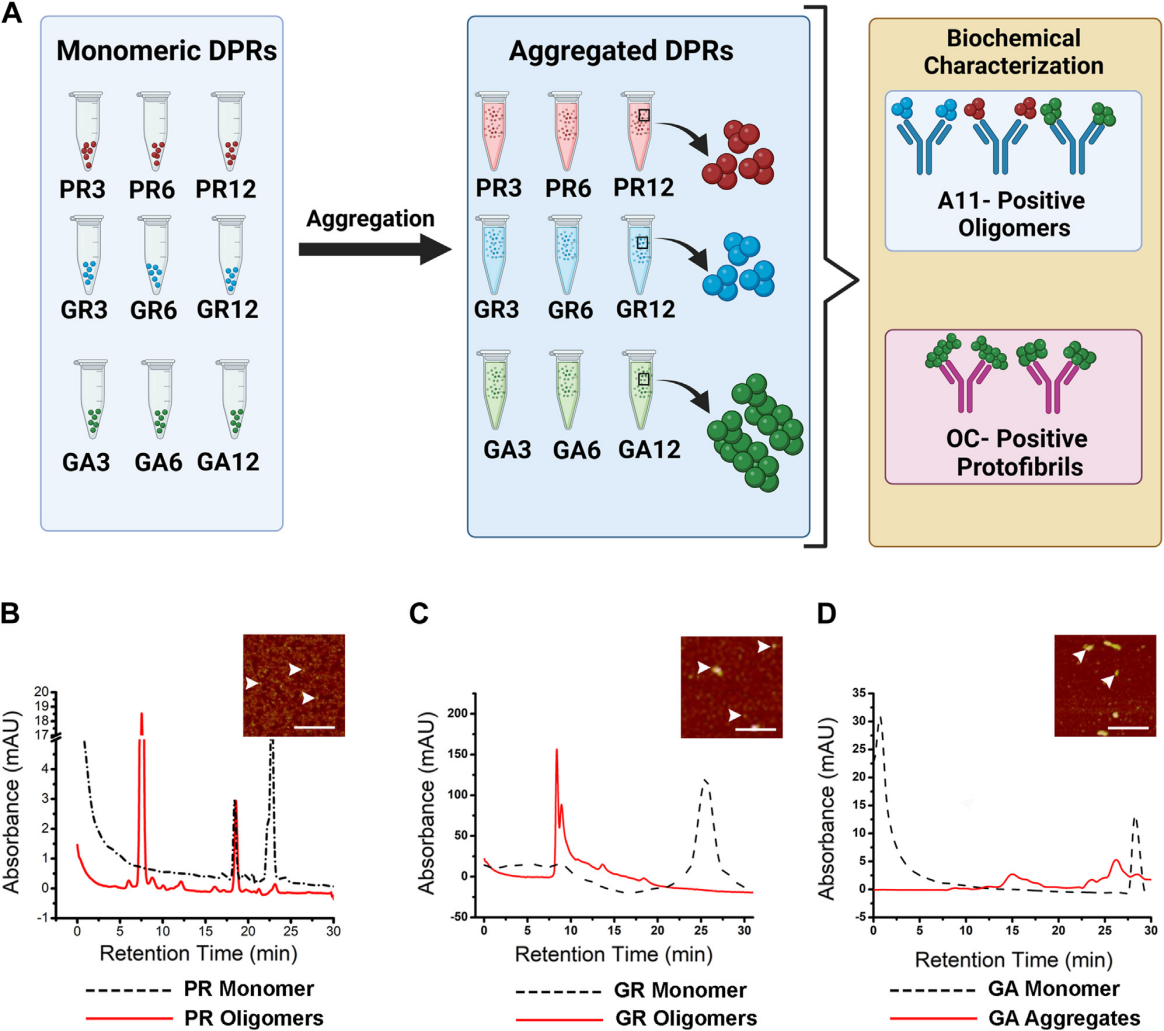
**Schematic representation of methodology and characterization**. *A*, diagram illustrating the aggregation process of DPRs, with GR depicted in *blue*, PR in *red*, and GA in *green*. GA is represented as a large filamentous shape followed by biological characterization using the oligomer-specific antibody A11–19 and fibril-specific antibody OC. *B*–*D*, size-exclusion chromatogram of PR, GR, and GA aggregates (*red line*) and respective monomers (*dashed line*). The signal was detected using 215 nm wavelength. AFM was also used to confirm aggregation (*inset*). DPR aggregates are indicated by *arrowhead*. Scale bar represents 100 nm. AFM, atomic force microscopy; DPR, dipeptide protein repeat; GA, glycine–alanine; GR, glycine–arginine; PR, proline–arginine.

**Figure 2:**
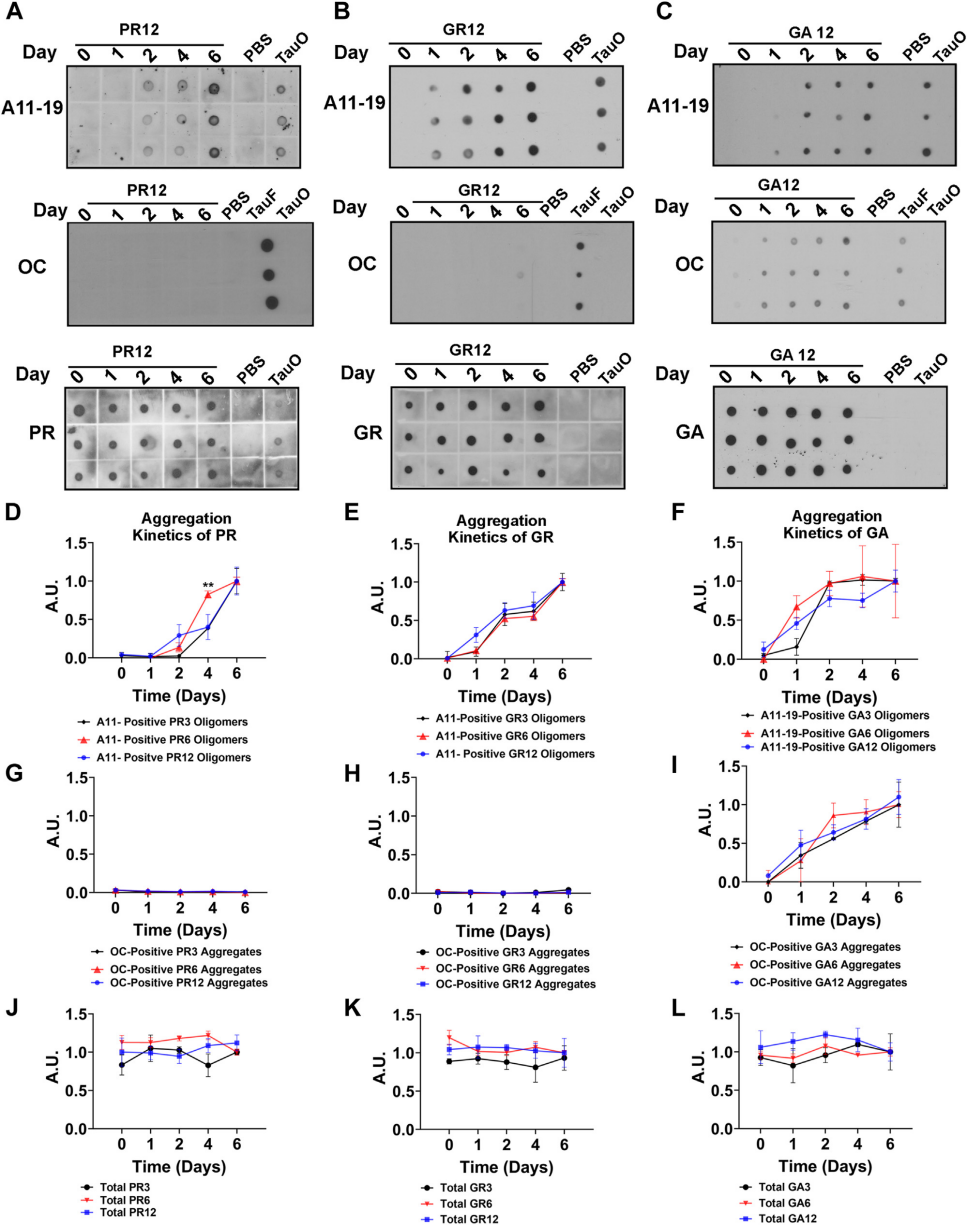
**The aggregation of GR and PR generates A11–19-positive oligomers, whereas GA generates OC-positive aggregates.***A* and *B*, aggregation of PR12 and GR12 was initiated by brief stirring, and samples were analyzed as a function of time by dot blot analysis. PR12 and GR12 samples were probed with oligomer-specific antibody, A11–19, and commercially available anti-PR and anti-GR antibodies, respectively. *C*, aggregation of GA12 was initiated by brief stirring, and samples were analyzed as a function of time by dot blot analysis. GA12 samples were probed with fibrillar conformation–specific antibody, OC, and commercially available anti-GA antibody. *D*–*L*, quantification of relative intensities of anti-PR, anti-GR, anti-GA, A11–19, and OC from DPRs with 3, 6, and 12 repeat lengths (representative dot blot images of three and six repeats are shown in Fig. S1). Analysis was conducted using one-way ANOVA with Tukey’s multiple comparisons test. Data are presented as mean ± SD. (∗∗*p* < 0.005 where n = 3). DPR, dipeptide protein repeat; GA, glycine–alanine; GR, glycine–arginine; PR, proline–arginine.

Several research groups, including Flores *et al*., have reported that DPRs form distinct structures and exhibit a unique cytotoxic effect depending on their repeat lengths. To investigate if the base unit was responsible for the formation of distinct cytotoxic amyloid oligomeric conformations, we employed a reductionist approach. Specifically, we utilized DPRs of varying lengths (3, 6, and 12) synthesized by Scenic Biotech. Immunological characterization of GR, PR, and GA aggregates with different repeat lengths was performed as illustrated in Figure 1*A*. In addition, we confirmed the aggregation of the DPRs using size-exclusion chromatography (SEC) (Fig. 1, *B*–*D*). In the aggregated GR samples, broad peaks were observed at shorter retention times when compared with the freshly prepared monomeric control. On the other hand, sharp peaks were observed at 7.5 and 18 min in the aggregated PR samples relative to the monomeric control. Similarly, broad peaks (15 min) were observed in the aggregated GA samples compared with the freshly prepared monomer control. Notably, the void volume excluded large peaks. Furthermore, we utilized atomic force microscopy (AFM) to confirm the aggregation of the DPRs (*inset*). The aggregates are indicated by *arrows* in the AFM inset. During the aggregation process of PR and GR, aliquots were taken daily and analyzed using dot blot, which were detected with the oligomer-specific antibody A11–19 (Fig. 2, *A*–*C*). Recombinant tau oligomers and PBS were used as positive and negative controls, respectively. The A11–19 antibody recognizes prefibrillar oligomers and generic conformational-dependent epitopes, which are not specific to a particular amino acid sequence (38, 39). As shown in Figures 2*A*, A11–19 was able to detect the presence of PR12 oligomers on day 2 of the aggregation process, with immunoreactivity increasing in a time-dependent manner (Fig. 2*A*). Similarly, GR12 oligomers were detected as early as day 1, and their formation continued to increase over time, as indicated by the increasing A11–19 immunoreactivity (Fig. 2*B*). Previous studies have reported that GA is highly prone to aggregation (15, 32). To further investigate the aggregation of GA12, we probed samples collected at different time points with the A11–19 antibody and observed the formation of A11–19 positive oligomers from the first day of aggregation, which persisted over the time frame investigated (Fig. 2*C*). Quantification of dot blots confirmed that both DPRs exhibited increased A11–19 immunoreactivity in a time-dependent manner (Fig. 2, *D*–*F*). These results suggest that PR may have slower aggregation kinetics compared with GR and GA (Fig. 2, *A*–*F*).

Interestingly, PR12 and GR12 demonstrated little to no immunoreactivity when probed with OC antibody (Fig. 2, *A* and *B*). This was also confirmed by the quantification of the dot blots (Fig. 2, *G* and *I*). The OC antibody recognizes a generic epitope that is associated with the fibrillar amyloid state, irrespective of the amino acid sequence (38, 39). This indicates that our PR12 and GR12 preparations did not form fibrillar aggregates that were detectable using OC within the time frame tested.

In addition, when probed with OC, GA12 showed time-dependent increased immunoreactivity, consistent with the formation of fibrillar aggregates (Fig. 2*C*). No immunoreactivity was observed when tau oligomer and PBS were probed with OC. Tau fibrils served as a positive control for OC. Furthermore, we used their respective commercially available DPR antibodies to confirm if the total amount of DRPs remained unchanged (Fig. 2, *J*–*L*). These findings suggest that, within the time frame investigated, PR12, GR12, and GA12 form oligomers that are positive for A11–19, whereas GA12 forms fibrillar aggregates that are positive for OC.

To determine whether this aggregation pattern is consistent across different repeat lengths, we also aggregated three and six lengths of DPRs (Fig. S1, *A*–*I*). Aliquots were collected daily, and dot blot analysis was performed, with membranes probed with A11–19 or OC. Similar to the 12-repeat length DPRs, GR and PR formed A11–19 positive oligomers after 1 and 2 days of aggregation and did not form OC-positive fibrillar aggregates (Fig. S1, *A*, *B*, *D*, and *E*). The same analysis was performed on GA3 and GA6 aggregates, and similar to GA12, the GA3 and GA6 aggregates also showed A11–19 immunoreactivity after the first day of aggregation. Moreover, the A11–19 immunoreactivity remained consistent over the investigated time frame (Fig. S1, *C* and *F*). We also investigated the presence of OC-positive fibrillar aggregates and observed that the immunoreactivity increased with time, similar to the GA12 repeats.

To further investigate the differences or similarities in the aggregation patterns between different DPRs and their repeat lengths, we conducted densitometric analysis (Fig. 2, *D*–*L*). As expected, GR and PR exhibited different aggregation kinetics. Interestingly, PR6 oligomers showed significantly higher (*p* < 0.005) immunoreactivity after 4 days of aggregation compared with the other repeat lengths, whereas there were no significant differences in different GR repeat lengths. Densitometric analysis of GA aggregates probed with OC showed that the immunoreactivity of GA3, GA6, and GA12 repeats increased with time, consistent with fibril formation (Fig. 2*F*). Overall, these findings suggest that each of the different DPRs have distinct aggregation kinetics, independent of repeat length.

### DPRs undergo secondary structure transition during aggregation

**Figure 3:**
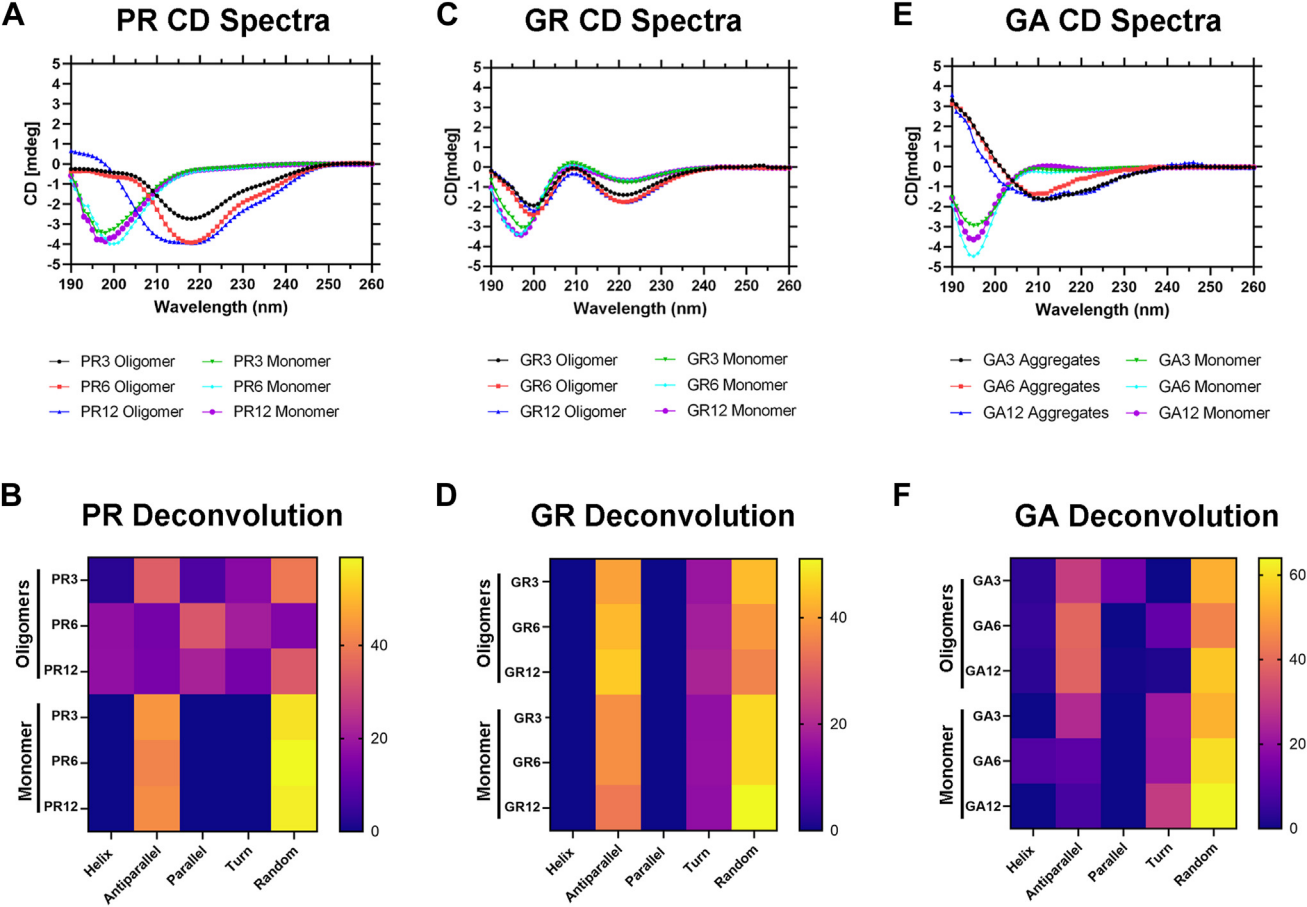
**DPRs undergo a secondary structure transition during aggregation.** Solution properties and CD deconvolutions of (*A* and *B*) PR oligomers, (*C* and *D*) GR oligomers, and (*E* and *F*) GA aggregates of 3, 6, and 12 repeat lengths. CD analysis was performed on the last day of the aggregation process. Respective monomers were used as controls for comparison. Deconvolutions were constructed using BeStel program and are represented as heat maps. DPR, dipeptide protein repeat; GA, glycine–alanine; GR, glycine–arginine; PR, proline–arginine.

Next, we assessed if DPRs undergo structural changes during the aggregation process. We performed far-UV CD spectroscopy on our DPR samples aggregated for 6 days and their respective monomers of each repeat length. The PR3 (*green line*), PR6 (*teal line*), and PR12 (*magenta line*) monomers show minima at 195 nm, reminiscent of disordered and flexible structures (Fig. 3*A*). The spectra were deconvoluted, which suggested that the monomeric PR structures were predominantly disordered. In contrast, the CD spectra of PR3 (*black line*), PR6 (*red line*), and PR12 (*blue line*) oligomers show broad minima at 215 nm to 220 nm, indicating a mixture of α-helixes and β-sheet secondary structures (Fig. 3*A*). This was also confirmed by the deconvolution (Fig. 3*B*). For GR, the spectra of monomeric GR3 (*green line*), GR6 (*teal line*), and GR12 (*magenta line*) indicated minima at 196 nm and a shallower minima 220 nm, whereas the oligomeric GR3 (*black line*), GR6 (*red line*), and GR12 (*blue line*) spectra showed sharp minima at 200 and 220 nm (Fig. 3*C*). The deconvolution indicated a transition from more disordered to a mixture of random coils and β-sheet secondary structures (Fig. 3*D*). The CD spectra of monomeric GA3 (*green line*), GA6 (*teal line*), and GA12 (*magenta line*) exhibited a minimum at 196 nm, similar to other disordered DPR monomers. Aggregated GA3 (*black line*), GA6 (*red line*), and GA12 (*blue line*) indicated a broad minimum at 210 nm (Fig. 3*E*). The deconvolution suggested a transition to a β-sheet secondary structure in the aggregated state (Fig. 3*F*).

### Arginine-containing DPR oligomers have distinct dye-binding properties and morphology

**Figure 4:**
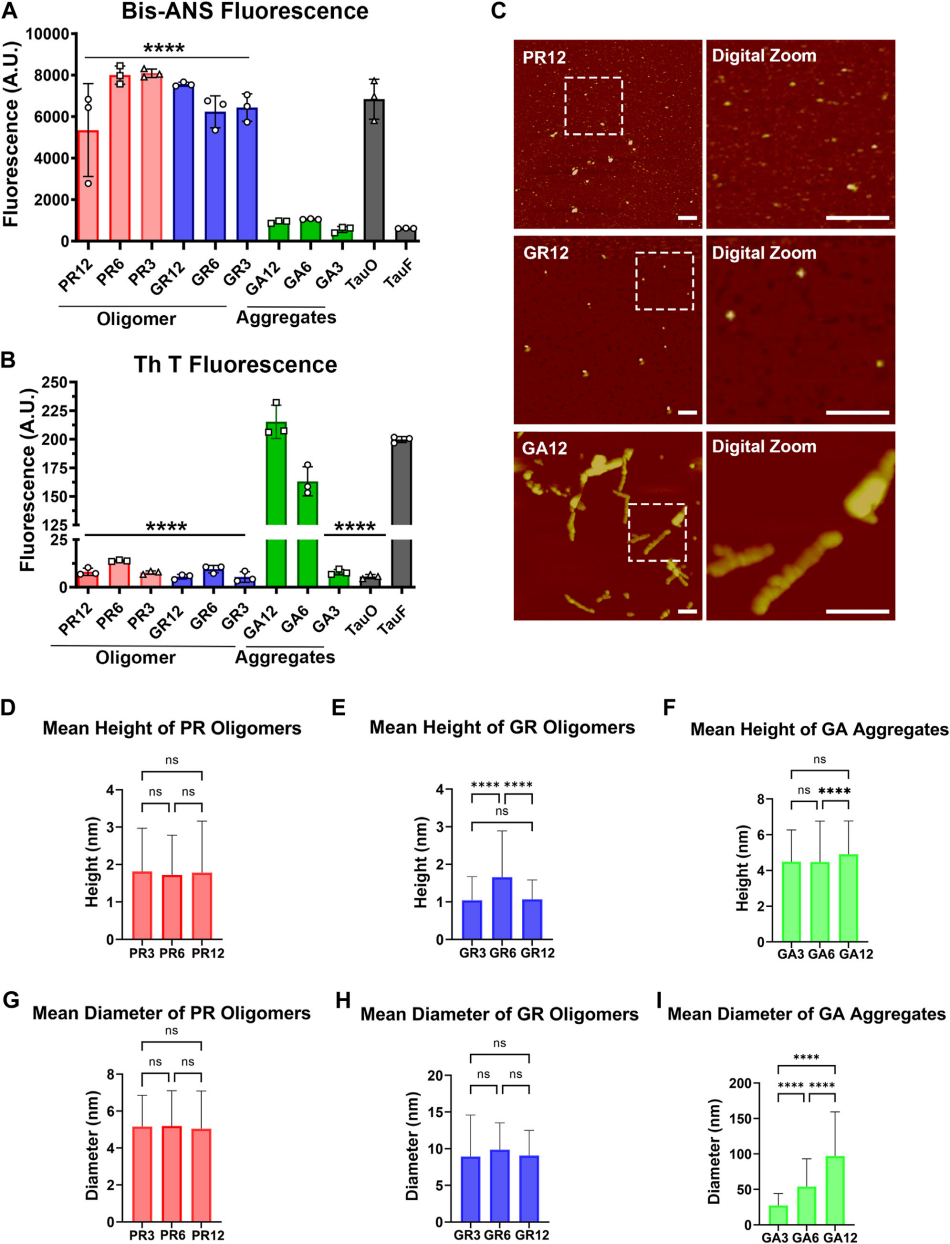
**GR and PR form small hydrophobic oligomers, whereas GA forms β-sheet-rich protofibrils.***A*, surface hydrophobicity was assessed using bis-ANS fluorescence dye for PR, GR, and GA aggregates of 3, 6, and 12 repeat lengths. PR and GR oligomers had significantly higher surface hydrophobicity compared with tau fibrils. *B*, β-sheet content was assessed using thioflavin T dye for PR, GR, and GA aggregates of 3, 6, and 12 repeat lengths. PR and GR aggregates show significantly less β-sheet content compared with tau fibrils (∗∗∗∗*p* < 0.0001). Tau oligomers and tau fibrils were used as negative and positive controls, respectively. *C*, representative AFM images of PR, GR, and GA aggregate on the sixth day of gentle stirring. Scale bar represents 100 nm. *D*–*I*, height and diameter distribution of PR, GR, and GA aggregates of 3, 6, and 12 repeat lengths. *D*–*I*, analysis was conducted using one-way ANOVA with Tukey’s multiple comparisons test. Data are presented as mean ± SD. (∗∗*p* < 0.005, ∗∗∗∗*p* < 0.0001); n = 3. AFM, atomic force microscopy; bis-ANS, 4,4′-dianilino-1,1′-binaphthyl-5,5′-disulfonic acid; GA, glycine–alanine; GR, glycine–arginine; PR, proline–arginine.

To investigate the structural characteristics of DPR aggregates associated with C9orf72, we performed thioflavin T (Th T) and 4,4′-dianilino-1,1′-binaphthyl-5,5′-disulfonic acid, dipotassium (bis-ANS) fluorescence binding assays to assess β-sheet content and surface hydrophobicity, respectively. Control experiments were performed using tau oligomers and fibrils. We found that GR and PR oligomers exhibited significantly higher surface hydrophobicity (*p* < 0.0001) compared with GA and tau fibrils, as evidenced by their increased binding to bis-ANS. This observation was consistent across all three different repeat lengths that were tested (Fig. 4*A*). On the other hand, the DPR monomers did not exhibit significant surface hydrophobicity compared with the tau fibrils (Fig. S2*A*). As expected, arginine-containing DPRs and tau oligomers exhibited weak binding to Th T compared with GA aggregates, which exhibited tight binding to Th T. This finding was consistent across all different repeat lengths tested (Fig. 4*B*). The DPR monomers did not exhibit significant binding to Th T compared with tau fibrils (Fig. S2*B*).

To further characterize the size and morphology of DPR aggregates, we used AFM. AFM images of aggregated PR12 and GR12 showed homogeneous spherical morphologies (Fig. 4*C*), similar to those observed for PR3, PR6, GR3, and GR6 (Fig. S2*C*). In contrast, GA12 exhibited distinct morphological differences, forming protofibrillar and fibrillar structures (Fig. 4*C*), which were similar to those observed for GA3 and GA6 (Fig. S2*C*). We also analyzed the abundance and distribution of different DPR species in the samples by plotting the height against the abundance from the AFM images using the particle analysis tool available in NanoScope Analysis software (Bruker). PR12 oligomers had an average height of 1.78 nm, which was consistent with PR3 and PR6 (1.81 and 1.72 nm, respectively) (Fig. 4*D*). GR3 and GR12 oligomers, regardless of repeat length, had an average height of 1.04 and 1.07 nm (Fig. 4*E*), respectively. Interestingly, GR6 oligomers exhibited a mean height of 1.6 nm, which was significantly higher than that observed for GR3 and GR12 oligomers. In contrast, GA12 protofibrils had an average height of 4.9 nm, which was similar to those observed for GA3 and GA6 protofibrils (4.48 nm and 4.47 nm, respectively) (Fig. 4*F*). To gain additional insight into the size of the aggregates, we analyzed the diameter of the DPR aggregates. The analysis of PR diameters of different repeat lengths revealed an average diameter of 5 nm (Fig. 4*G*). GR of different repeat lengths exhibited mean diameters of 9 nm, whereas GA12 aggregates were significantly larger (*p* < 0.0001), with an average diameter along their major axis of 97 nm. GA6 and GA3 aggregates were slightly smaller, with average diameters of 54 nm and 27 nm, respectively (Fig. 4*I*).

Taken together, our results indicate that PR and GR form small unique oligomeric structures that are more hydrophobic and have low β-sheet content compared with GA aggregates. This distinction was observed across all the different repeat lengths that were tested.

### DPRs oligomers are found in ALS and FTD postmortem brain tissue

**Figure 5:**
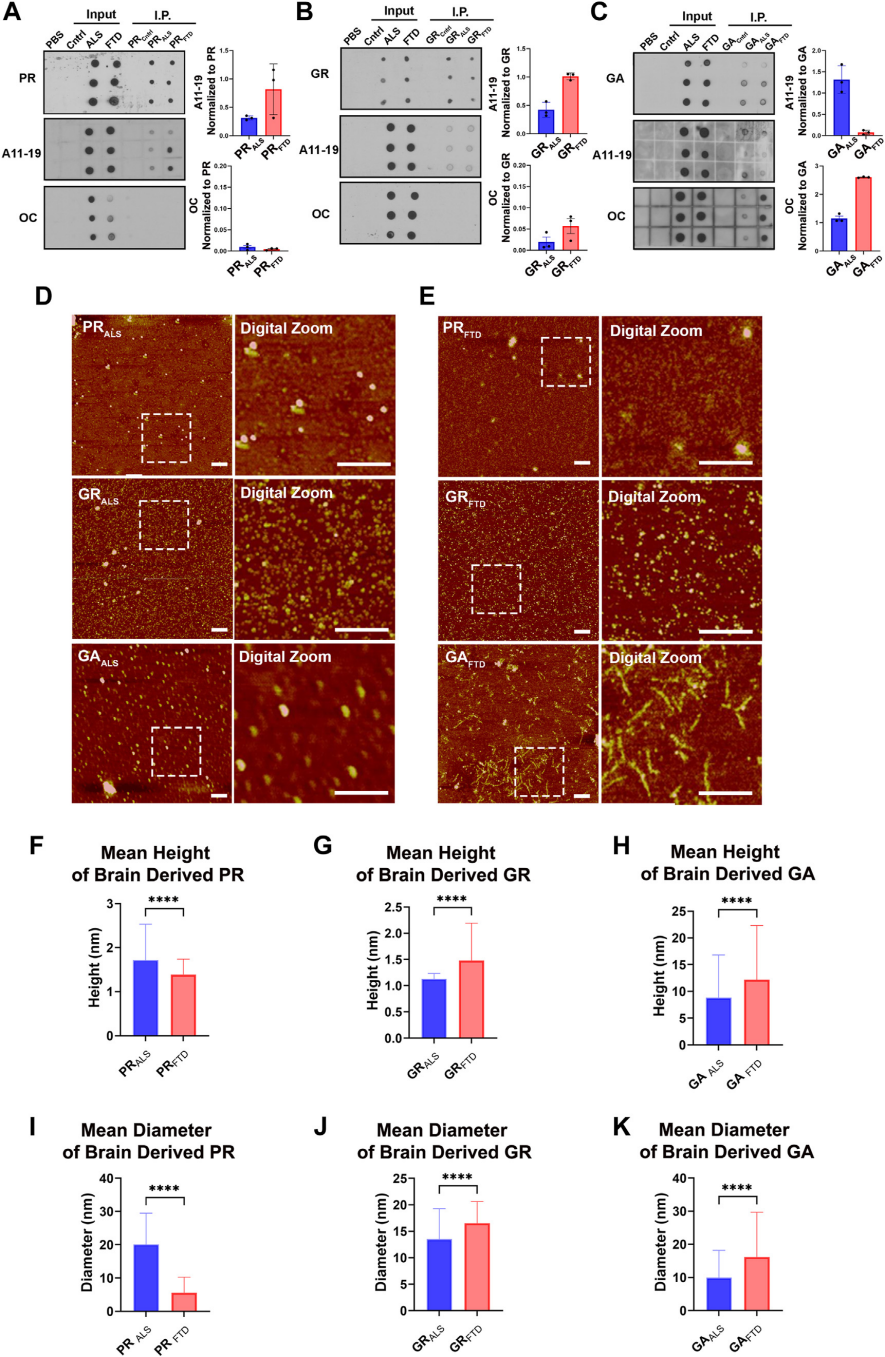
**ALS and FTD postmortem brain tissues have A11–19-positive PR, GR, and GA oligomers as well as OC-positive GA protofibrils.***A*, dot blot analysis of brain-derived DPRs immunoprecipitated with PR, (*B*) GR, and (*C*) GA repeats probed with oligomer-specific A11–19 antibody, fibrillar-specific OC antibody, and their respective commercial DPR antibodies. *D* and *E*, representative AFM images of DPRs immunoprecipitated from ALS and FTD postmortem brain tissues. Scale bar represents 100 nm. *F*–*H*, height and (*I*–*K*) diameter distribution of DPRs immunoprecipitated from ALS and FTD brain tissues. Unpaired Student’s *t* test was conducted. Data are presented as mean ± SD. (∗∗*p* < 0.005, ∗∗∗∗*p* < 0.0001). ALS, amyotrophic lateral sclerosis; DPR, dipeptide protein repeat; FTD, frontotemporal dementia; GA, glycine–alanine; GR, glycine–arginine; PR, proline–arginine.

As demonstrated in the aforementioned data, DPRs, particularly GR and PR, form oligomers *in vitro*. We then further investigated whether DPR oligomers are found in ALS and FTD postmortem brains. Brain homogenates from ALS, FTD, and age-matched controls were prepared and subjected to immunoprecipitation using commercial antibodies specific for PR, GR, and GA. Immunoreactivity for each of the DPR antibodies was observed in ALS and FTD samples but not in the age-matched controls. To determine whether the immunoprecipitated brain–derived DPRs were oligomeric or fibrillar, we evaluated their immunoreactivity to the oligomer-specific A11–19 or protofibril-specific OC antibodies. A11–19 immunoreactivity was detected in the ALS and FTD samples for PR, GR, and GA, indicating the presence of oligomers in the brain-derived DPR samples. Minimal to no immunoreactivity to the OC antibody was observed in brain-derived PR and GR samples, whereas strong immunoreactivity to OC was observed in ALS- and FTD-derived GA, indicating the presence of GA protofibrils/fibrillar oligomers (Fig. 5, *A*–*C*). The quantification of each conformational antibody, normalized to their respective commercially available DPR antibodies, is presented on the right.

To further validate the morphology of DPRs isolated from ALS and FTD patients, we performed AFM followed by quantification of their average height distribution and diameter. PR_ALS_ formed compact and spherical oligomers, whereas PR_FTD_ showed diffuse, small, and spherical oligomers (Fig. 5, *D* and *E*). However, PR_ALS_ and PR_FTD_ had significantly different average height (*p* < 0.0001), 1.7 and 1.3 nm, respectively (Fig. 5*F*). PR_ALS_ had an average diameter of 20 nm, significantly larger than PR_FTD_ (*p* < 0.0001), which had an average diameter of 5.2 nm (Fig. 5*I*). ALS- and FTD-derived GR had similar compact, small, spherical oligomers, but GR_FTD_ appeared more heterogeneous (Fig. 5, *D* and *E*). The heterogeneity was also identified by the height distribution, as GR_ALS_ had an average height of 1.1 nm and GR_FTD_ had an average height of 1.4 nm (Fig. 5*G*). GR_ALS_ and GR_FTD_ oligomers showed an average diameter of 13.4 nm and 16.5 nm, respectively (Fig. 5*J*). However, the brain-derived GA showed diverse morphologies in both the ALS and FTD samples. GA_ALS_ aggregates had a kidney bean–like morphology, whereas GA_FTD_ appeared protofibrillar (Fig. 5, *D* and *E*). The height distribution of GA from ALS and FTD showed that the aggregates were much larger compared with brain-derived PR and GR oligomers, with an average height of 8.4 nm and 12.16 nm, respectively (Fig. 5*H*). Moreover, GA_FTD_ had a significantly larger height (*p* < 0.0001) compared with GA_ALS_. In addition, the average diameter for GA_ALS_ and GA_FTD_ was 9.0 nm and 16.13 nm, respectively (Fig. 5*K*).

Overall, our data from immunological and morphological analysis suggest that brain-derived PR and GR form oligomers, whereas GA aggregates from ALS and FTD brains are larger and appear fibrillar. Interestingly, the brain-derived DPRs were morphologically similar to the DPR aggregates generated *in vitro*. These findings provide insight into the pathogenesis of ALS and FTD.

### The oligomeric conformations of DPRs are toxic in SH-SY5Y neuroblastoma cells

**Figure 6:**
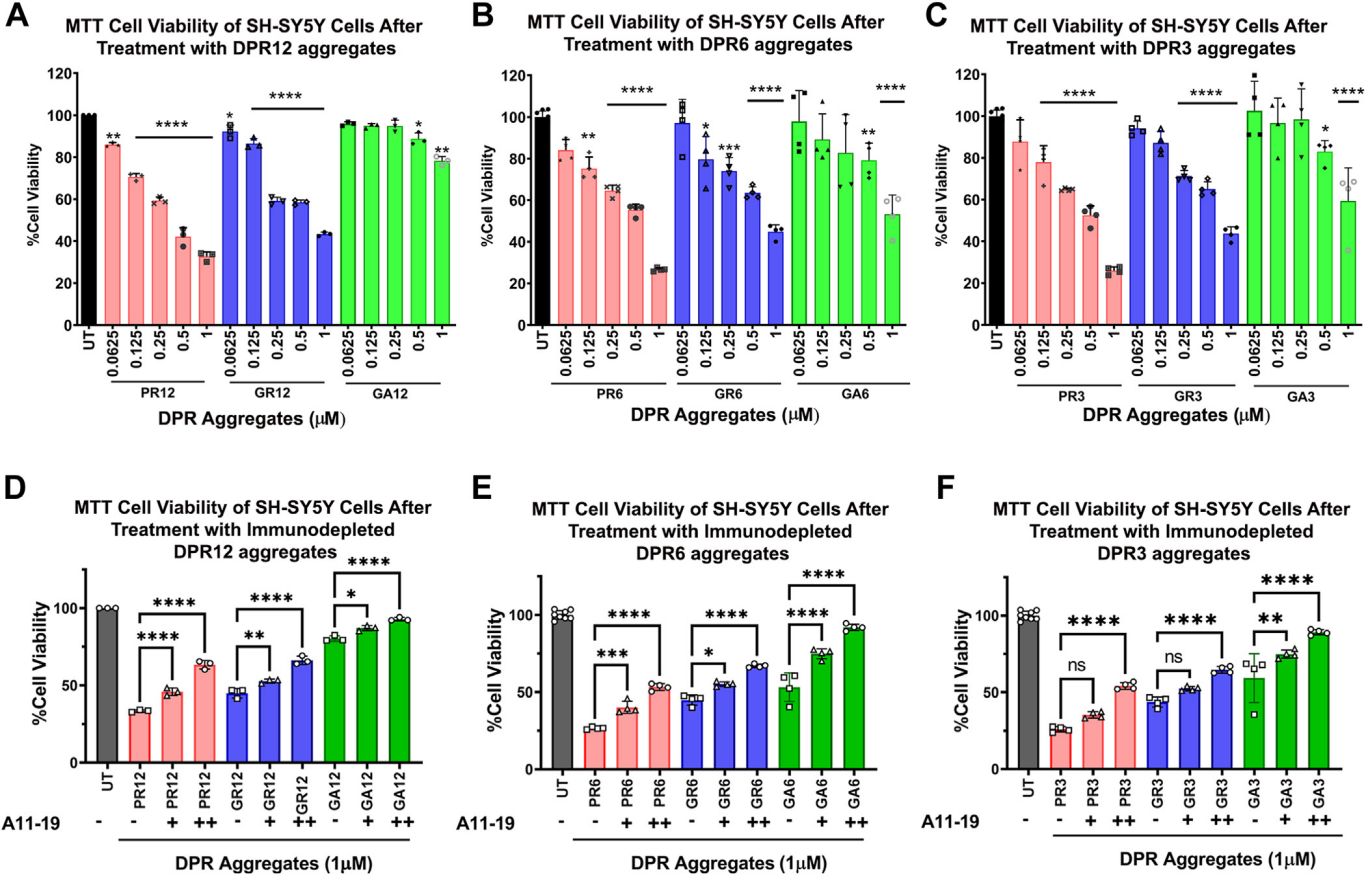
**Repeat length–independent toxic DPR oligomers can be modulated by oligomer-specific antibody neutralization.***A*–*C*, cell viability assay of SH-SY5Y cells treated with 0.0625 to 1 μM of different DPR aggregates with 3, 6, and 12 repeat lengths. *D*–*F*, DPR aggregates with or without preincubation with the oligomer-specific antibody A11–19 at a 1:2 ratio (+) or 1:4 ratio (++) for 1 h. The data demonstrate that PR and GR oligomers are significantly cytotoxic compared to the untreated. The toxicity of PR and GR oligomers is significantly reduced upon preincubating them with affinity-purified A11–19 antibody. The GA ensemble exhibited significantly greater toxicity compared with UT control cells; however, this toxicity was ameliorated upon preincubation with affinity-purified A11–19 antibody. Analysis was conducted using one-way ANOVA with Tukey’s multiple comparisons test. Data are presented as mean ± SD. (∗*p* < 0.05, ∗∗∗*p* < 0.005, ∗∗∗∗*p* < 0.0001). DPR, dipeptide protein repeat; GA, glycine–alanine; GR, glycine–arginine; PR, proline–arginine.

Since we identified the presence of DPR oligomers in ALS and FTD, we asked the question if the oligomeric conformation was toxic in a cellular model. As reported by Westergard *et al*. (40), DPRs can be transmitted cell to cell. White *et al*. (41)have also reported that PR causes toxicity by disrupting nucleolar function when applied exogenously. We treated SH-SY5Y neuroblastoma cells with DPR oligomers and measured the cell viability after 24 h. As shown in Figure 6*A*, the GR12 and PR12 oligomers significantly reduced the cell viability of SH-SY5Y in a dose-dependent manner, whereas the GA12 ensemble was only significantly toxic at higher concentrations. We also evaluated the cell viability of SH-SY5Y after treatment with three and six repeats, where GR and PR oligomers with three and six repeats showed a significant reduction in cell viability, whereas the GA aggregates of three and six repeats were significantly toxic at higher concentrations (Fig. 6, *B* and *C*).

To determine whether the DPR oligomers were responsible for the observed toxicity, we treated the cells with DPRs preincubated with affinity-purified oligomer-specific antibody A11–19 for 1 h. Our results demonstrated that the preincubation of increasing amounts (1:2, 1:4 protein to antibody) of A11–19 antibody reduced the toxicity observed by both GR12 and PR12 oligomers (Fig. 6*D*). This was consistent across different repeat lengths tested (Fig. 6, *E* and *F*). In addition, preincubation of increasing amounts of A11–19 antibody decreased the cytotoxicity of GA12 aggregates (Fig. 6, *D*–*F*). These findings suggested that neutralizing the oligomers reduced the cytotoxic effects of DPRs.

To investigate whether the fibrillar conformation was responsible for triggering the observed toxicity, we treated DPRs alone and preincubated with affinity-purified OC antibody (1:4 M ratio). Our results showed that the preincubation with OC antibody did not rescue the cell viability for PR12, GR12, and GA12 (Fig. S3*A*), and this pattern was consistent among the different repeat lengths (Fig. S3, *A*–*C*).

We treated cells with aggregates formed by seeding 12-repeat DPR with brain-derived material. We confirmed the oligomerization after the seeding processing using dot blot analysis (Fig. S4*A*). We treated SH-SY5Y cells with brain-derived DPRs alone or preincubated with increasing amounts (1:2, 1:4 protein to antibody). Our results showed that the brain-derived DPRs were toxic, where ALS- and FTD-derived PR showed the most reduction in cell viability. Moreover, preincubation with the A11–19 antibody significantly increased the cell viability observed for brain-derived PR and GR (Fig. S4, *B* and *C*). Brain-derived GA was significantly toxic, and there was not a significant increase in cell viability after preincubation with A11–19.

Taken together, our findings suggest that the different DPR oligomers exhibit varying degrees of toxicity in a cellular model, and that the oligomeric conformation, rather than the fibrillar conformation, is responsible for the observed toxicity. In addition, neutralizing the oligomers with A11–19 antibody reduces the cytotoxic effects of DPRs. These results provide insight into the toxic mechanism of DPRs and may facilitate the development of therapeutic strategies for ALS and FTD (Figs. 6, S3, and S4).

## Discussion

In this study, we found that like many other amyloidogenic proteins, soluble oligomers of C9orf72-associated DPRs are the primary cytotoxic species. The aim of the study is to build on the work of Flores *et al.* (32), which is twofold: first, to show that DPRs form A11-positive oligomers *in vitro*, as well as can be found in the brain and second, to demonstrate their cytotoxic effects independent of repeat length.

Previous studies have shown that DPRs are toxic and can inhibit vital cellular mechanisms (19, 20, 26, 28). However, most of these studies use various repeat lengths that show similar toxic effects. Flores *et al*. (32) showed that sense RNA RAN-translated GR, GA, and GP form distinct structures, which they call “spherical aggregates.” They further showed that GR and GA aggregates are neurotoxic (32). Seeing this, we show that the DPRs may take a unique metastable conformation, independent of their repeat length and thus understanding this conformation will be an important step in understanding toxic mechanisms of DPRs. Here, we aggregated the most toxic DPRs (both sense and antisense) in the literature, PR, GR, and GA of three different repeat lengths. The DPRs formed A11–19-positive aggregates. Previous observations with the A11–19 antibody suggest that it recognizes a conformation that is distinct from that of soluble monomers, large insoluble aggregates, and fibrils, suggesting that DPRs may form this conformation as well. We leveraged the conformational detection of A11–19 to understand the aggregation kinetics of the DPRs. PR12 and GR12 had different aggregation kinetics, where GR12 oligomers were detectable earlier than PR12 oligomers. This was similar, if not the same across the other two repeat lengths tested. As for GA, previous studies have reported that it exhibits a high propensity for aggregation and forms fibrils more rapidly than GR or PR (15). As such, we probed it with a fibril-specific antibody, OC. The OC antibody suggests that it recognizes a conformation that is distinct from that of soluble monomer or oligomers. Fibrillar GA12 was detectable as early as the first day of aggregation and increased as a function of time. A similar pattern was observed with GA6 and GA3 repeats. Biochemically, GR, PR, and GA are amyloids that have low complexity in their amino acid sequences and are aggregation prone (15, 16). Like with other amyloid proteins such as amyloid β, tau, and α-synuclein, DPRs undergo a structural change as they aggregate. In the case of PR, there was a stark change in the secondary structure, as the monomeric PR showed mostly random coils that transitioned to a mixture of α-helices and β-sheet structures as it aggregated. Interestingly, α-helices may be a common secondary structure in proline-rich proteins (42). GR, on the other hand, showed an increase in β-sheets as it transitioned from monomer to oligomeric structures. A β-sheet secondary structure was also visible with GA aggregates, which was consistent with other published reports (15, 32).

The DPR aggregates also had distinct surface hydrophobicity and β-sheet content using dye-binding assays. Generally, oligomers of amyloidogenic proteins have high surface hydrophobicity compared with their fibrillar counterparts (43, 44). In the case of DPRs, PR and GR had high surface hydrophobicity and low β-sheet content compared with GA, which had high β-sheet content. AFM of the DPRs showed that PR and GR formed small spherical oligomers, whereas GA was more protofibrillar, which explained the strong Th T signal as well as the OC immunoreactivity. PR and GR oligomers were smaller in comparison to oligomers of other proteins (30, 31, 45). The small size is expected since we have used short repeat lengths. The GA ensemble exhibited a combination of fibrillar oligomers and protofibrils, distinguishing itself with the largest height and diameter compared with the various DPRs tested. This was also consistent with the reports published by Flores *et al.* (32).

Since the discovery of RAN translation and DPRs in ALS and FTD brains, most studies have focused on *in vitro* and *in vivo* models to understand different aspects of DPR-mediated toxicity (18). In this report, we immunoprecipitated DPRs from ALS and FTD postmortem brain tissue, allowing us to understand the biochemical and morphological characteristics that may be present in ALS and FTD brains. The immunoprecipitated samples showed A11–19-positive signal from all brain-derived DPRs, suggesting the presence of PR, GR, and GA oligomers. When we tested the extracted samples against OC, brain-derived PR and GR showed a sparse, if not completely absent, signal. Strong OC immunoreactivity was observed in brain-derived GA, suggesting the presence of protofibrils and fibrillar oligomers, which were not observed in brain-derived PR and GR samples. AFM further validated these results. PR_ALS_ and PR_FTD_ oligomers were spherical and compact but were morphologically distinct. The GR_ALS_ and GR_FTD_ appeared morphologically similar. Interestingly, GA_ALS_ and GA_FTD_ were morphologically different and were larger than brain-derived PR and GR. GA_FTD_ appeared to have an ensemble or mixture of fibrillar oligomers and protofibrils. We identified A11–19-positive PR and GR oligomers and OC-positive protofibrils in ALS and FTD brains, which have been unprecedented in the field thus far.

Finally, a simple cell viability assay showed that the DPR oligomers generated are toxic. Furthermore, when the DPR-aggregated samples were preincubated with oligomer-specific antibody, A11–19, their toxicity was reduced. In essence, the antibody bound to the oligomers and neutralized their toxicity, which was consistent across all the repeat lengths. Previous reports have shown that PR and GR are cytotoxic, whereas the GA-mediated cytotoxicity is controversial (16, 18, 26). Our reports indicate that residual GA oligomers are toxic, and when they are neutralized with A11–19, their toxicity is reduced. The cell viability did not increase when DPRs were preincubated with the fibril-specific antibody OC. Moreover, we leveraged prion-like spreading properties of amyloids and seeded brain-derived DPRs to monomeric DPRs with 12 repeats. Amyloid seeds imprint their properties on the native monomers, creating conformations that resemble the original seeds (46, 47, 48, 49, 50). Brain-derived DPRs were toxic, and preincubation with A11–19 increased the cell viability. This, irrevocably, showed that the oligomer conformation of all DPRs play a significant role in toxicity, more so than their repeat lengths. The observations reported resemble other neurodegenerative diseases like Alzheimer’s and Parkinson’s diseases where amyloid-β, tau, and α-synuclein oligomers are the most toxic (51, 52, 53). Like amyloid-β, tau, and α-synuclein, DPRs undergo structural changes, particularly in regard to their secondary structure, surface hydrophobicity, and size. These characteristics are vitally important in understanding the toxicity of amyloids (44). In the case of amyloid-β and α-synuclein, toxicity appears to be independent of secondary structure (54). However, this correlation appears unclear with other amyloidogenic proteins, and especially with DPRs, as PR and GR formed a mixture of secondary structures and were the most toxic. Surface hydrophobicity may have a direct connection with toxicity, as protein oligomers interact with membranes leading to ion dyshomeostasis (44, 55, 56). This may explain the increased toxicity that is observed in the hydrophobic PR and GR oligomers but not in large GA aggregates. Furthermore, size plays an important role in amyloid internalization and toxicity (43), also explaining how PR and GR oligomers may be more toxic compared with GA aggregates.

The reductionist approach used here aids our understanding of the structure and oligomeric conformations of DPRs. It is increasingly evident that among the various factors that contribute to neurodegeneration, further investigation on oligomer-induced toxicity could prove beneficial. This study isolates DPR oligomers from ALS and FTD postmortem brain tissues and characterizes them morphologically. This link between structural characteristics of DPR oligomers and toxicity suggests that the oligomer species may be a key contributor of the disease. The translation of this knowledge can be put into therapeutic strategies, which would impact not only just ALS and FTD but also other neurodegenerative diseases.

## Experimental procedures

### Generating DPR aggregates

DPRs of three different lengths, 3, 6, and 12, were synthesized by Scenic Biotech. DPR aggregates were prepared as detailed previously (32). In brief, the DPRs were solubilized in 1× PBS and were gently stirred for 6 days. Aliquots were taken at days 1, 2, 4, and 6 for analysis. Samples were aliquoted and stored until further use.

### Generation of tau oligomers and fibrils

Tau 2N4R monomer was expressed recombinantly and purified as described previously (57, 58, 59). Aliquots of monomeric tau (1 mg/ml) were incubated with heparin (15 kDa) at a 1:4 ratio in 1× PBS for 5 days (59). The samples were rotated constantly using an orbital shaker at 30 RPM. The tau oligomers were generated by orbital shaking of the tau monomer (0.6 mg/ml) for 48 h in sterile 1× PBS at room temperature. The resulting tau oligomers were purified by fast liquid chromatography (Superdex 200 increase 10/300; Cytiva) using 1× PBS as a mobile phase at 0.75 ml/min (58).

### SEC

SEC was used to validate the aggregation. About 10–20 μg of protein was loaded on a Superdex 75 increase using a 20 μl loop. 1× PBS was used as the mobile phase at a flow rate of 0.75 ml/min, and the signal was detected using a wavelength of 215 nm.

### Dot blot analysis

Each sample was diluted to 1 mg/ml prior to use. About 1 μl of sample was dotted onto a 0.2 mm nitrocellulose membrane and then blocked with 10% nonfat milk in Tris-buffered saline with 0.01% Tween (TBS-T). The membranes were then probed with the previously characterized oligomer-specific A11–19 antibody (1:8000 dilution) or fibril-specific OC (1:10,000 dilution) antibody for 1 h at room temperature (4, 30, 39). Commercially available PR (Millipore; catalog no.: ABN13454) (1:1000 dilution), GR (Millipore; catalog no.: ABN1360) (1:1000 dilution), and GA (Millipore; catalog no.: MABN889) (1:1000 dilution) antibodies were used for their respective membranes. Membranes were then washed three times with TBS-T and incubated with the respective horseradish peroxidase–conjugated immunoglobulin G anti-rabbit (1:10,000 dilution) and antimouse (1:10,000 dilution) secondary antibodies. The blots were washed three times in TBS-T, and ECL plus (GE Healthcare) was used for signal detection. Densitometric analysis of each band was carried out using ImageJ (National Institute of Health) and analyzed by two-way ANOVA followed by multiple comparisons test using Prism 9.1 (Graphpad).

### Bis-ANS and ThT fluorescence

Samples were prepared by adding 0.6 μg of protein and 248 μl of 10 mM bis-ANS, in 100 mM glycine–NaOH buffer (pH 7.4), in a clear bottom 96-well black plate. Each experiment was performed in triplicate. The excitation and emission wavelengths used to measure fluorescence intensity were 380 nm and 520 nm, respectively.

For ThT assay, samples were prepared using 0.6 μg of protein and 248 μl of 5 mM ThT, 50 mM glycine–NaOH buffer (pH 8.5). Each experiment was performed in triplicate. The fluorescence intensity was measured at an excitation wavelength of 440 nm and an emission wavelength of 490 nm using a POLARstar OMEGA plate reader (BMG Labtechnologies).

Fluorescence spectra of Bis-ANS and ThT + vehicle were used to correct for the background fluorescence.

### CD

Jasco-720 (Jasco, Inc) equipped with a temperature controller was used to measure the CD spectra of the samples. A scan speed of 20 nm/min at 0.2 nm interval was used to record the spectra. Protein concentration of 0.1 mg/ml was used in a quartz cell of 1 mm pathlength. The spectra were measured in 1× PBS (pH 7.4) from 195 to 250 nm with an average of three iterations used for each spectrum. After every sample, the quartz cell was washed with water and ethanol between every use. The spectra were analyzed using K2D3 software (60) and plotted using OriginPro 8.5 (OriginLab). The spectra were deconvoluted using the BeStSel algorithm (61).

### AFM

DPR oligomer preparations were imaged by AFM using a noncontacting tapping method with a multimode 8 AFM machine (Bruker). In brief, 1 μl of each sample was diluted in 9 μl of molecular grade water (corning) and was applied to freshly cleaved mica surface and allowed to be absorbed at room temperature overnight. Mica was then washed with 200 μl of water and air dried. Images were taken from a minimum of five different areas on the mica surface. AFM images were analyzed by using particle analysis tool from NanoScope Analysis v1.20rl AFM data processing software to assess the height distribution of each sample.

### Brain homogenate preparation

Postmortem brain tissue from ALS and FTD carrying the C9orf72 HRE were obtained from Michigan University Brain Bank. The neuropathological assessment conformed to the National Institute on Aging/Reagan Institute consensus criteria. The homogenization of postmortem brain tissue was conducted as mentioned previously (62). Briefly, each brain was homogenized in lysis buffer (50 mM Tris–HCl, pH 7.6, 150 mM NaCl, 0.01% NP-40, 2 mM EDTA, and 0.1% SDS) with protease inhibitor Cocktail (Roche; catalog no.: 11836145001), using 1:3 dilution of brain:lysis buffer (w/v). Samples were centrifuged at 10,000 RPM for 10 min at 4 °C. The supernatants were aliquoted, snap frozen, and stored at −80 °C until use.

### Immunoprecipitation of DPRs from human brain tissues

Immunoprecipitation of DPR was performed using Pierce coimmunoprecipitation (ThermoFisher; catalog no.: 26149) according to the manufacturer’s instructions. In brief, the coupling resin was incubated with 75 μg of PR, GR, or GA antibody. The antibody-coupled resin was incubated with brain homogenate of ALS and FTD cases overnight at 4 °C. The following day, the flowthrough was collected, the resin was washed using the manufacturer-provided wash buffer. The DPRs were eluted using 0.1 M glycine (pH 2.4). The samples were stored at −80 °C until further use.

### Amplification of brain-derived DPRs

The brain-derived DPRs were amplified using monomeric DPRs with 12 repeats. Brain-derived DPRs were incubated using a 1:70 M ratio (seed to monomer). The samples were then rotated on an orbital shaker at 37 °C for 72 h (48, 49, 59, 63). The aggregation was confirmed using dot blot analysis.

### Immunodepleting and cell viability assay

SH-SY5Y neuroblastoma cells were maintained using Dulbecco’s modified Eagle's medium with 10 mM Hepes, 10% fetal bovine serum, 4 mM glutamine, penicillin (200 U/ml), and streptomycin (200 μg/ml) in 5% CO_2_ at 37 °C. The medium was replaced every 2 days, and cells were plated in a 96-well plate having 8000 cells per well and grown overnight. The medium was removed, and various concentrations (0.0625 to 1 μM) of PR, GR, and GA preparations were applied to the cells. Separately, PR, GR, and GA preparations were preincubated with affinity-purified A11–19 for 1 h at a 1:4 ratio (protein:antibody) before treatment to cells. The cells were incubated for 6 h at 37 °C and then were subjected to MTT colorimetric assay using Cell Proliferation I (MTT) assay kit (Roche) with the manufacturer’s instructions. Each treatment was performed in triplicates, and one-way analysis of variance was used for statistical analysis.

## Data availability

All the data are present with this article.

## Supporting information

This article contains supporting information and is available at the *Journal of Biological Chemistry*’s website. The datasets used during the current study are available from the corresponding author on reasonable request.

## Conflict of interest

The authors declare that they have no conflicts of interest with the contents of this article.

## Figures and Tables

**Suplemental Figure 1:**
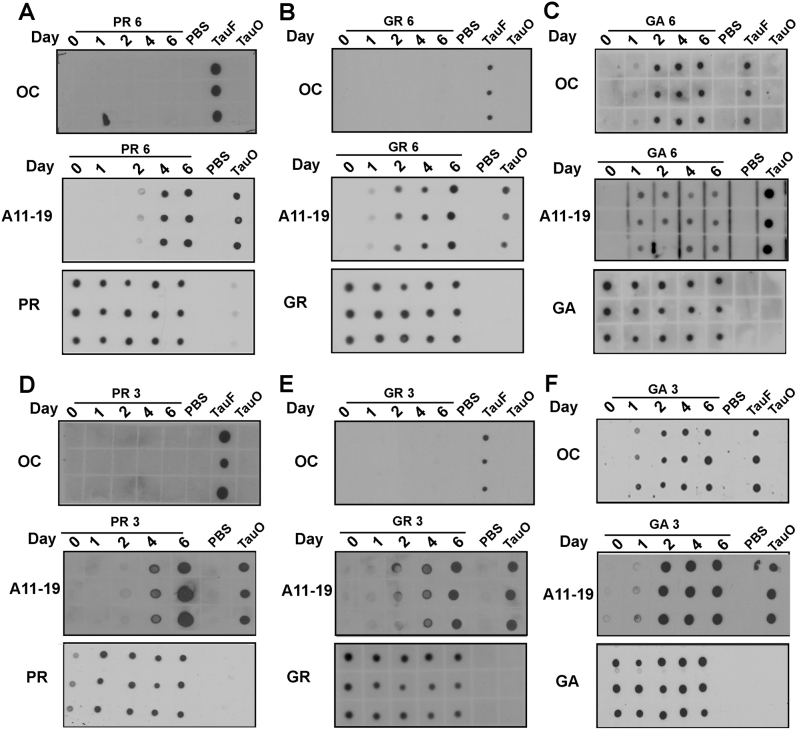


**Suplemental Figure 2:**
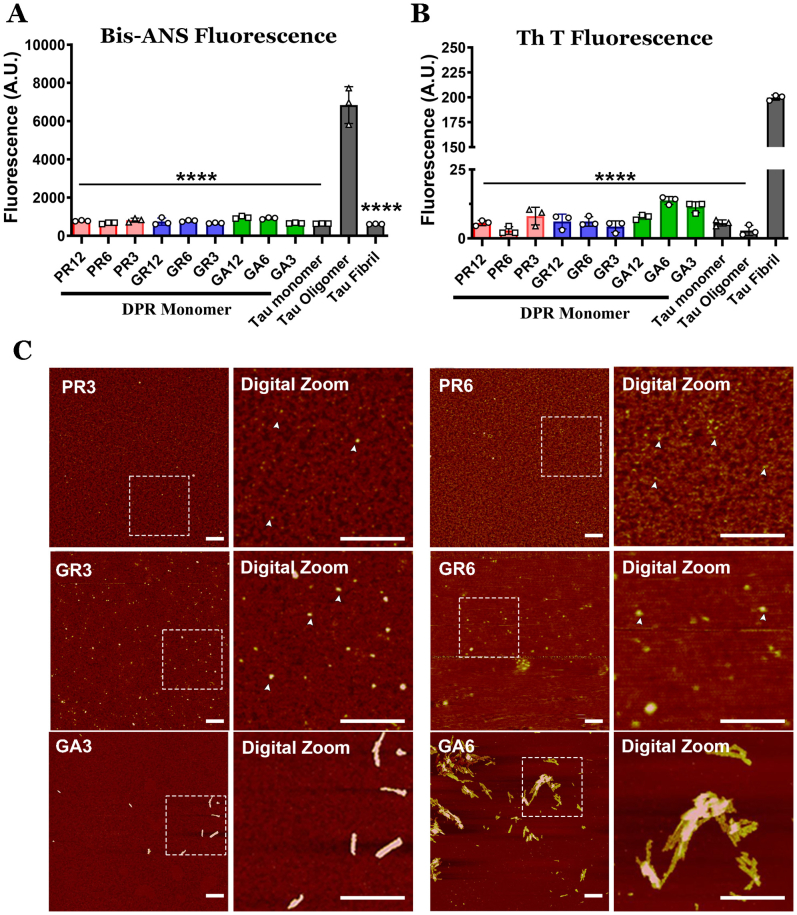


**Suplemental Figure 3:**
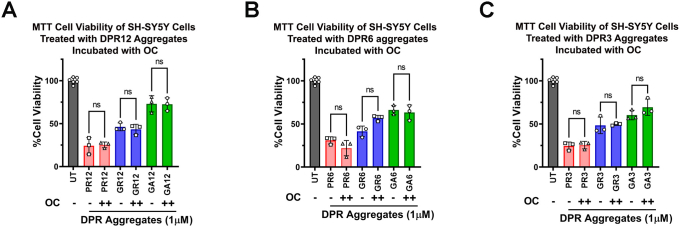


**Suplemental Figure 4:**