# Supplementary material for: C9orf72-associated dipeptide protein repeats form A11-positive oligomers in amyotrophic lateral sclerosis and frontotemporal dementia
Source: J Biol Chem. 2024 Jan 10;300(2):105628. doi: 10.1016/j.jbc.2024.105628 (PMC10844744; doi:10.1016/j.jbc.2024.105628)
Supplement: Supplemental Figure Legends [file mmc1.docx]

**Supplemental Figures**

**Figure S1: PR and GR form A11-19 positive oligomers while GA forms OC positive aggregates independent of repeat lengths. (A-C)** Dot blot analysis of PR, GR6, and GA6 using fibril specific antibody, OC, oligomer specific antibody, A11-19 and total DPR antibodies. PR12 and GR12 did not produce any OC positive aggregates during the investigated timeframe. (**D-F**) Dot blot analysis of DPRs with 3 repeats immunoblotted with OC, A11-19, and their respective DPR antibodies. GR and PR with 3 and 6 repeats generate A11-19 positive aggregates but do not form OC positive fibrillar aggregates. Conversely, GA with 3 and 6 repeats form aggregates that are detected by both A11-19 and OC.

**Figure S2. Monomeric DPRs have distinct dye-binding properties and DPR aggregates have unique morphologies independent of repeat lengths. (A)** Surface hydrophobicity is assessed using bis-ANS fluorescence dye for PR, GR, and GA monomers with 3, 6, and 12 repeat lengths. **(B)** β-sheet content was assessed using Thioflavin T dye for PR, GR, and GA monomers with 3, 6, and 12 repeat lengths. Tau oligomers and fibrils were used as controls (****P<0.0001). **(C)** Atomic Force Microscopy (AFM) analysis of DPRs of 3 and 6 repeat lengths**.** AFM of PR3, PR6, GR3, GR6, GA3, and GA6 aggregates after 6 days of gentle stirring. Scale bar = 100nm. Analysis was conducted using one-way ANOVA with Tukey’s multiple comparisons test. Bars and error bars represent mean values and standard deviations, respectively.

**Figure S3. Preincubation of DPR aggregates with OC does not increase cell viability, while brain derived DPRs can seed, and their cytotoxicity can be neutralized by A11-19**. **(A-C)** MTT cell viability of SH-SY5Y cells treated with DPR aggregates of different repeat lengths incubated with OC at a 1:2 ratio (+) or 1:4 ratio (++). Preincubation of DPRs with OC does not increase cell viability, indicating that the fibrillar DPR conformation is not cytotoxic. Analysis was conducted using one-way ANOVA with Tukey’s multiple comparisons test. Bars and error bars represent mean values and standard deviations, respectively.

**Figure S4. Brain derived DPRs can seed, and their cytotoxicity can be neutralized by A11-19**. (**A**) Confirmation of brain derived DPR seeding was assessed through dot blot analysis using A11-19, OC and their respective DPR antibodies. Seeding with brain derived DPRs generated A11-19 positive oligomers. Of note, OC positive aggregates were also detected in GA. (**B,C**) MTT cell viability of SH-SY5Y cells treated with ALS and FTD derived DPR aggregates alone or pre-incubated with A11-19 at 1:2 ratio (+) or 1:4 ratio (++) for 1h. Preincubation of DPRs with A11-19 significantly increased (**P<0.005, ****P<0.0001) the cell viability. Analysis was conducted using one-way ANOVA with Tukey’s multiple comparisons test. Bars and error bars represent mean values and standard deviations, respectively.
